# Development, implementation and outcome of standards to restrict fatty meat in the food supply and prevent NCDs: learning from an innovative trade/food policy in Ghana

**DOI:** 10.1186/1471-2458-14-249

**Published:** 2014-03-13

**Authors:** Anne Marie Thow, Reginald Annan, Laudina Mensah, Syeda Nafisa Chowdhury

**Affiliations:** 1 Menzies Centre for Health Policy, School of Public Health, University of Sydney, Victor Coppleson Building (D02), Sydney NSW 2006, Australia; 2 Department of Biochemistry and Biotechnology, College of Science, Kwame Nkrumah University of Science and Technology, Kumasi, Ghana

**Keywords:** Fatty meat, Non-communicable disease, Policy analysis, Ghana, Trade policy

## Abstract

**Background:**

Diet-related noncommunicable diseases represent a major global public health challenge, and require a multisectoral policy response. However, the use of trade policy in this context has met with varied success in the face of strong global trade liberalization agendas. The Government of Ghana has implemented an innovative food standards policy to limit the amount of fat in meat and meat cuts, in response to rising imports of low quality fatty meat cuts. This paper presents an analysis of the policy process and outcomes, as well as contextual factors in policy development, to enable policy learning in other jurisdictions.

**Methods:**

We conducted 28 semi-structured policy analysis interviews with 37 stakeholders at the national and regional level in Ghana, and collated relevant documents. We analysed the data using the health policy analysis triangle and policy theories related to lesson drawing.

**Results:**

The standards were developed in response to health concerns related to fatty meat (particularly turkey tails), in a context of rising meat imports and a generalised concern about the low quality and high fat content of imported meats. The standards were the result of collaboration between the trade and health sectors. The standards apply to both imported and domestic meat, and were designed to be compliant with Ghana’s multilateral trade commitments. The overall effect of the ban has been to reduce availability of specific ‘low quality’ high-fat meats in the Ghanaian food supply, namely turkey tails and chicken feet.

**Conclusions:**

This study indicates that the use of standards can reduce availability of high-fat meat in a national food supply. The main strength of a standards approach to reducing fatty meat (mainly imported) in the food supply is compliance with global trade law, while the main challenge is effective enforcement. However, the Government of Ghana appears to have developed a functional and flexible application of the policy. Features of this policy approach useful for policy learning include: collaboration at every stage between ministries of trade and health; considerations relating to compliance with international trade law; strategic enforcement of the policy; and the importance of public awareness efforts.

## Background

Diet-related noncommunicable diseases (NCDs) represent a major global public health challenge [1]. Even in Sub-saharan Africa, a region still beset by high rates of undernutrition and communicable diseases, the prevalence of diabetes, cancer and heart disease represent a significant burden of disease [2]. These diseases have a complex causality: changing food supplies, urbanization, increasing life expectancy and sedentary work and leisure activities are giving rise to new patterns of eating and activity. There is thus a need for innovative upstream policy approaches to improve diets and prevent NCDs by targeting the ‘causes of the causes’ [3]. The multisectoral approach recommended by the World Health Organization’s 2013–2020 global action plan includes the use of policies from finance, trade and agriculture [4].

As part of this multisectoral approach, international institutions, non-government agencies and academics have recommended trade policies designed to improve the healthfulness of the food supply and prevent NCDs [5-7]. Trade in foods is one of the upstream drivers of global dietary change; it has been associated with shifts to diets high in fat, sugar and salt, associated with NCDs [8,9]. In particular, the importation of cheap high-fat cuts of meat has been identified in Africa and the Pacific as a contributor to both NCDs and agricultural under-development [10,11].

Policies in the Pacific banning imported fatty meat products have met with varied success in the face of strong global trade liberalization agendas [12]. Requirements for non-discrimination in trade agreements limit the scope for countries to reduce availability of specific (imported) products. For example, Samoa was required to remove a four-year ban on turkey tail imports as a condition of their World Trade Organization (WTO) accession in 2011 [13]. As an alternative option, the use of standards to reduce the availability of high-fat meat products based on their fat content have been proposed, but not evaluated [14]. This approach to improving the food supply has several potential benefits over product-specific bans, including reducing the likelihood of replacement with other fatty meat products, being non-discriminatory in its application to both imported and domestic products, and automatically applying to new/novel meat cuts or products (without requiring amendments to legislation).

Ghana implemented an innovative food standards policy to limit the amount of fat in beef, mutton, pork and poultry in response to rising imports of low quality meat with liberalization of trade in the early 1990s. This appears to be the only policy of its kind globally, but the development, implementation and effect of this policy has not yet been assessed. This paper presents an analysis of the policy process and outcomes of this innovative trade policy intervention to improve diets. It also identifies contextual factors in policy development, to enable policy learning in other jurisdictions.

## Methods

We used case study research and policy analysis methodologies to evaluate the policy, which enabled in-depth assessment of the policy process and outcomes in Ghana, while maximising usefulness of the case study for policy learning in other contexts [12,15,16]. We used the ‘health policy analysis triangle’ as the overarching framework for the research, which identifies content, context, process (apexes of the triangle) and actors (in the centre of the triangle), as well as the interaction between these four elements, as critical for understanding health policy making [17,18]. Based on this framework, we collected data on the *process* (agenda-setting, policy development, implementation), *context*, *content* and *actors* (local and international) for the policy, as well as the policy outcomes, guided by research questions based on the policy process (the relevant additional dimensions from the framework are shown in brackets):

– What was the nature of the policy intervention? (content)

– Why was it proposed? (actors, context)

– How did it get onto the political agenda? (context, actors)

– Who is responsible for implementing the policy, and how has it been implemented? (content, actors)

– What was the outcome of the policy initiative?

We drew on both qualitative and quantitative sources to answer these questions, including interviews (with policy makers, implementers, producers, processors and retailers), trade data (local import data, FAOSTAT trade data from the United Nations Food and Agriculture Organization, export data from major source countries), food availability and production data (FAOSTAT and local data), information on trade policy change (policy and national plan documents from the Government of Ghana; WTO trade policy reviews) and information on food supply policies (Euromonitor; United States Department of Agriculture Foreign Agricultural Service reports on policy and markets). We also triangulated interview data through analysis of documents relevant to the case study policy, identified through interviews, internet searches, and hard-copy searches of major newspapers for the year 1999 [15]. These included food standards, newspaper reports, internal government and industry background papers, and public commentary.

We identified stakeholders for interview by sending letters of invitation to the heads of the key policy and implementing agencies with relevance to the fatty meat restriction, as well as to the CEOs of several leading meat importers and agricultural producers. All government agencies contacted agreed to allow a representative(s) to participate, as did the majority of companies contacted. Further relevant stakeholders within Government agencies were also identified via snowball sampling during interviews, but interviewees did not indicate that we should contact further agencies.

We conducted 28 semi-structured interviews with 37 stakeholders in Accra and Kumasi in August 2013 (AMT, RA, LM attended all interviews), from the following groups: Policy makers in Trade (4), Health (4) and Agriculture (4); Implementers in Ghana Standards Authority (2), Ghana Food and Drugs Authority (2), Animal Extension (1) and Customs Excise and Preventive Service (2); and Agricultural producers, Traders and importers (8). As outlined above, we asked specifically about the process and outcome of the restrictions on fatty meat, and also asked broad contextual questions regarding the historical trade policy context, changes in food prices and agricultural production, and the perceived effect of food imports on agricultural production. We used iterative analysis throughout the interviewing to identify and pursue themes based on the research framework. Detailed notes from all interviews were recorded by two interviewers. Within one day of all interviews, they were transcribed and sent to the interviewee for checking and amendment.

We present our analysis using the health policy analysis triangle. The themes identified were also informed by policy theories related to lesson drawing, in order to inform the capacity for policy learning in other contexts [19-21]. These include: the type of actors and their role in the process; the structure and content of the policies and the instruments selected; the political dynamics and processes involved; and the interaction with global factors and actors.

This study was approved by the Committee on Human Research, Publications and Ethics, Kwame Nkrumah University of Science and Technology, School of Medical Sciences and Komfo Anokye Teaching Hospital, Kumasi, Ghana.

## Results and discussion

### Content

The percentages for fat content of pork, beef, mutton and poultry meat and meat cuts are found in standards that have been published by the Ghana Standards Authority (GSA, previously the Ghana Standards Board) These specify that carcasses and cuts of pork and beef shall contain no more than 25% fat, poultry no more than 15% fat and lamb no more than 30% fat (detail in Table 1).

**Table 1:** Text of standards relating to fat in meat

**Type of meat**	**Relevant text in food standards**
Pork	‘Deboned carcasses/cuts (minus the backfat) shall contain not more than 25% total fat when determined in accordance with clause 3 of GS 70 [this is the standard that describes how to measure fat in meat]. Backfat thickness: pork carcass shall have a backfat thickness not exceeding 2.5 cm’, 2008 [22]
Beef	‘Deboned carcasses and cuts shall contain not more than 25% fat by mass when determined in accordance with clause 3 of GS 70’, 2003 [23]
Mutton	‘Mutton carcases/cuts excluding the back fat shall contain not more than 25% fat by mass. Where the back fat is not removed a maximum of 30% fat by mass shall be permitted, when determined in accordance with clause 3 of GS 70’, 2005 [24]
Poultry	‘Dressed poultry and/or poultry parts shall have fat content of not more than 15 percent when determined in accordance with clause 3 of GS 70’, 2003 [25]

Source: Ghana Standards Authority Library, Accra, Ghana.

While these standards are quite recent, it is evident from interviews and documentation (e.g. World Trade Organization Trade Policy Reviews) that these standards regarding fat content also appeared in the first edition of the standards, which are no longer available (due to being superseded by the second edition). The original standards were implemented in the early 1990s. There is a discrepancy between the percentages of fat given in the GSA documents, and those in the 2001 and 2008 WTO Trade Policy Reviews, which cite the figures as 25% for beef, 42% for pork, 35% for mutton, and 15% for poultry [26,27]. For the purposes of this paper, we will refer to the standards contained in the second edition of the GSA documents as the relevant standards, as we were unable to determine the reason for this discrepancy.

### Context

The key contextual factors that appear to have influenced the development of the standards were health concerns related to fatty meat, rising meat imports, and the status of Ghana as a member state of the General Agreement on Tariffs and Trade (GATT, precursor to the WTO).

NCDs began to rise in Ghana during the 1980s. By 1991 cardiovascular disease was the leading cause of death, and the diabetes prevalence in urban areas was 2–3% [28]. Ghana is one of the few countries in Africa to have a well-established ‘nutrition transition’, and has relatively low infant mortality rates, high levels of obesity/overweight, and low levels of underweight in women [29]. A survey on animal source food consumption in the early 1990s found that fish was consumed daily by the majority of people, usually as part of soups or stews, while meat was eaten on average two to three times a week [30]. At the time, fish was the cheapest source of protein available. Fresh and frozen meat is the main type of meat consumed in Ghana. Tinned and processed meats are available, but comprise a small component of production, imports and consumption.

**Figure 1:**
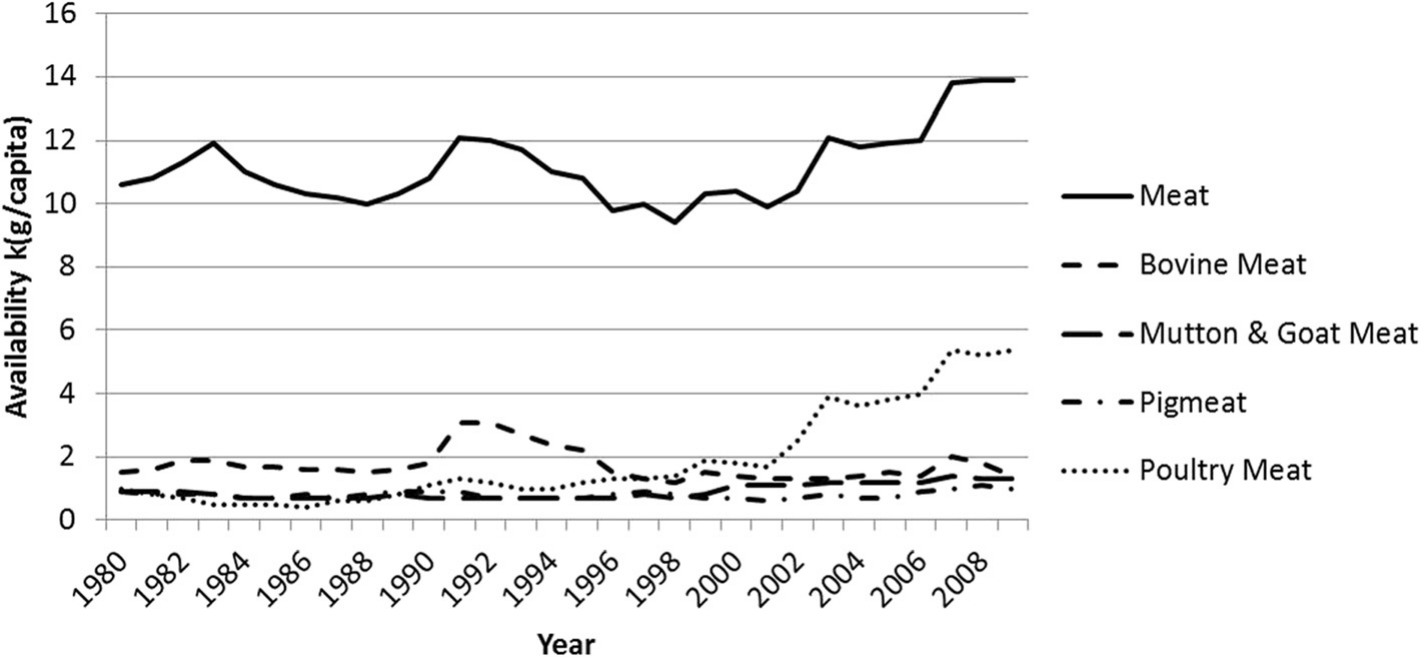
**Availability of meat in Ghana, 1980–2009.** Source: Food and Agriculture Organization, FAOSTAT Food Balance Sheet data (http://faostat3.fao.org/faostat-gateway/go/to/home/E).

Per capita meat availability in Ghana has increased from around 11 kg in 1990 to 14 kg in 2009 (Figure 1). In the 1970s and 1980s, the domestic industry supplied around 90% of the meat consumed, mainly by large livestock farms in Northern Ghana, and substantial poultry farms in the middle belt of Ghana, but by the 1990s supplied only around 35%.

Ghana joined the GATT after independence in 1957, but followed a protectionist policy regarding agriculture until the mid-1980s. Due to the implementation of Structural Adjustment Programs as conditions to World Bank and IMF loans, and to the ongoing Uruguay Round negotiations, Ghana began to implement policies of liberalization, with the progressive removal of import licensing and tariff reductions for the majority of imports from 1989 [31-33].

**Figure 2:**
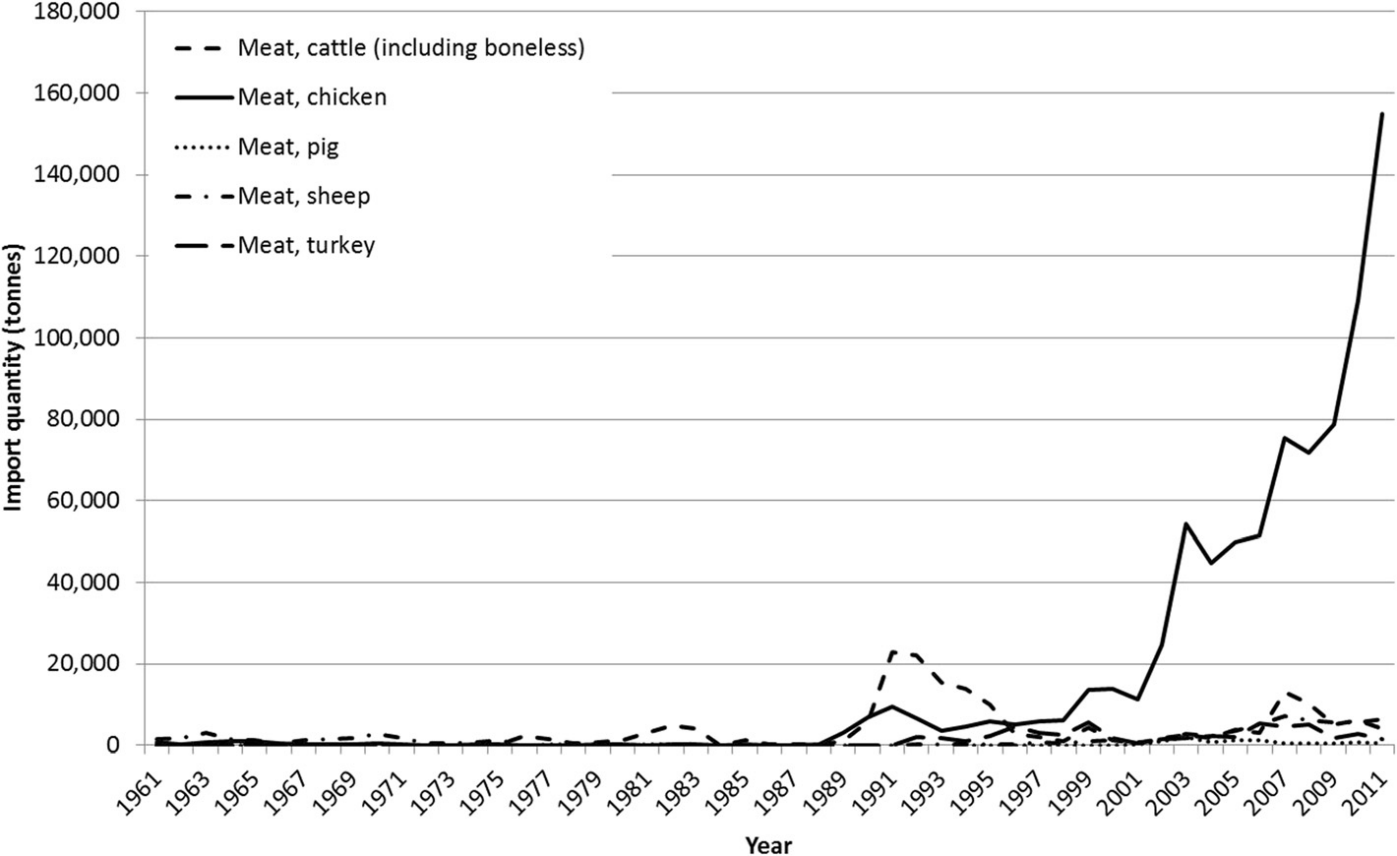
**Imports of meat in Ghana, 1961–2011.** Source: Food and Agriculture Organization, FAOSTAT trade data (http://faostat3.fao.org/faostat-gateway/go/to/home/E).

Meat imports began to rise in the late 1980s and early 1990s with introduction of these policies to promote trade liberalization. Meat imports have increased dramatically, particularly imports of poultry cuts (Figure 2). Poultry has historically been imported from the United States of America (USA) and European Union (EU), but since the mid-2000s the share of imports from Brazil has increased. The Netherlands, the USA and Brazil are currently the three main source countries for poultry imports [34].

### Process

#### Agenda setting

Interviewees reported that it was mainly concern over high fat/low quality poultry meats (and particularly turkey tails, ‘tsofi’ (around 32-40% fat [fresh] [35])) that led to the development of the standards, but that this was part of a generalised concern about the low quality and high fat content of imported meats. The focus of concern was population health, and the Ghana Health Service and Ministry of Health played a key role in identifying the need for the standards. Interviewees reported that there was public concern about high-fat diets (particularly saturated fat) in relation to rising rates of NCDs. This seemed to be part of a broader perception that imported meat was of low quality; interviewees commented that it was common for imported meat to be stored for a long time before sale, that imported meat often appeared to have been ‘coloured’, and that the imported cuts of meat had a low protein content and were generally ‘off-cuts’ rather than the whole carcass (as one interviewee put it: ‘They send us the tails… but where is the turkey?!’). There was no mention of protection of domestic agriculture as a reason for the standards.

#### Development

The standards were developed by the GSA, then the Ghana Standards Board. Interviewees reported that the standards were the result of collaboration between the Ministries of Trade, Health and Agriculture, and that the Ministry of Trade issued the directive for their development (GSA is an implementing agency of the Ministry of Trade). The technical committees that developed the standards included representatives from GSA, FDA, Ministries of Health, Trade and Agriculture, the Council for Scientific and Industrial Research, universities, and other agricultural research institutes.

Percentages of fat given in the standards were based on the association of high-fat meat consumption with NCDs, particularly cardiovascular disease, and the cut-offs for what constitutes 'high-fat' were derived from analysis of the fat content of local and imported meats. Interviewees reported that they represent a ‘reasonable’ expectation of the amount of fat in meat and meat cuts. The standards apply to both imported and domestic meat, and are thus non-discriminatory. Interviewees from the Ministry of Trade reported that membership of the GATT/WTO influenced the choice of a non-discriminatory and evidence based standard for reducing fatty meat imports.

#### Implementation and enforcement

The implementation and enforcement of the standards are currently the responsibility of the Ghana Food and Drugs Authority (FDA, previously the Food and Drugs Board, which is an implementing agency of the Ministry of Health), which was established in 1992. Meat is on the ‘high risk imports’ list, which means that the GSA takes a sample of any meat entering the country for testing in relation to a wide number of different quality standards. The focus of this testing is on quality from a micro-biological perspective. However, the implementing officers also consider standards compliance more broadly. The GSA notifies the FDA where there is a need for enforcement. The FDA also conducts spot checks locally, at cold stores (mainly frozen imported meat) and butchers (mainly local meat). All local meat sold must be killed in an abattoir, and these are also subject to inspection. Prior to importation, meat traders must have FDA approval of their facilities and obtain a permit from Veterinary Services (Ministry of Agriculture). However, the application for the permit does not require a declaration of the percentage of fat in the intended import.

All importers interviewed were aware of the restrictions on meat imports related to fat. One industry interviewee related how, when applying for a loan for his importing business, he was told that importing turkey tails was not allowed. The butchers that we interviewed were not aware of the standards, but reported that consumers requested that the fat be trimmed from the meat before weighing and purchase. There was no public awareness campaign to accompany the ban.

The initial focus of the ban was on turkey tails, but in the late 1990s medical specialists noticed a high availability of chicken feet in Ghana, which they were concerned about because of the lack of bioavailable protein and presence of high amounts of fat in this ‘cut’. They lobbied the Ministry of Trade to use the standard to restrict their import. In response, the Ministry of Trade issued a press release in September 1999 stating that chicken feet were not permitted to be imported under the standard. This press release also contained a reminder to importers of the restrictions on fat in meat, and stated that these standards would be enforced [36]. While the usual percentage of fat in chicken feet (around 15% fat [after boiling] [37]) appears to be close to the fat content permitted, it may be that the chicken feet that were being imported had a higher percentage of fat since fat in poultry varies depending on feed, cooked weight and other variables (the authors were unable to obtain estimates of the fat content of the imports of concern).

Imports of turkey tails rose again in the late 2000s, generating public commentary regarding the low quality of the meat and the need for enforcement in 2009–2010 and again in 2012 [38,39]. In response, the FDA issued press releases about the health effects of diets high in saturated fat (in relation to turkey tail consumption), and increased enforcement of the ban, destroying a large number of imported turkey tails [40-42].

Almost all interviewees reported that it is still possible to obtain turkey tails in the market, generally imported over the land border with Togo, but that the amount available was very low compared to what it was before the standards were implemented.

### Actors

A notable aspect of the actors involved was the interplay between health and trade at all stages: agenda setting, development, implementation, and enforcement all involved collaboration between the Ministries of Health and Trade and their agencies (FDA and GSA at latter phases).

There was, however, a dearth of actors at the international level. Two interviewees reported that the USA had suggested removal of the standard, and one additional interviewee attributed this to the World Bank, but there was no evidence of contest through formal channels (a search of the WTO and GATT notifications and complaints). Similarly, there was no mention of international agencies influencing the agenda setting process, other than the contextual effect of WTO membership in informing the choice of a non-discriminatory and justifiable tool.

### Outcome

**Figure 3:**
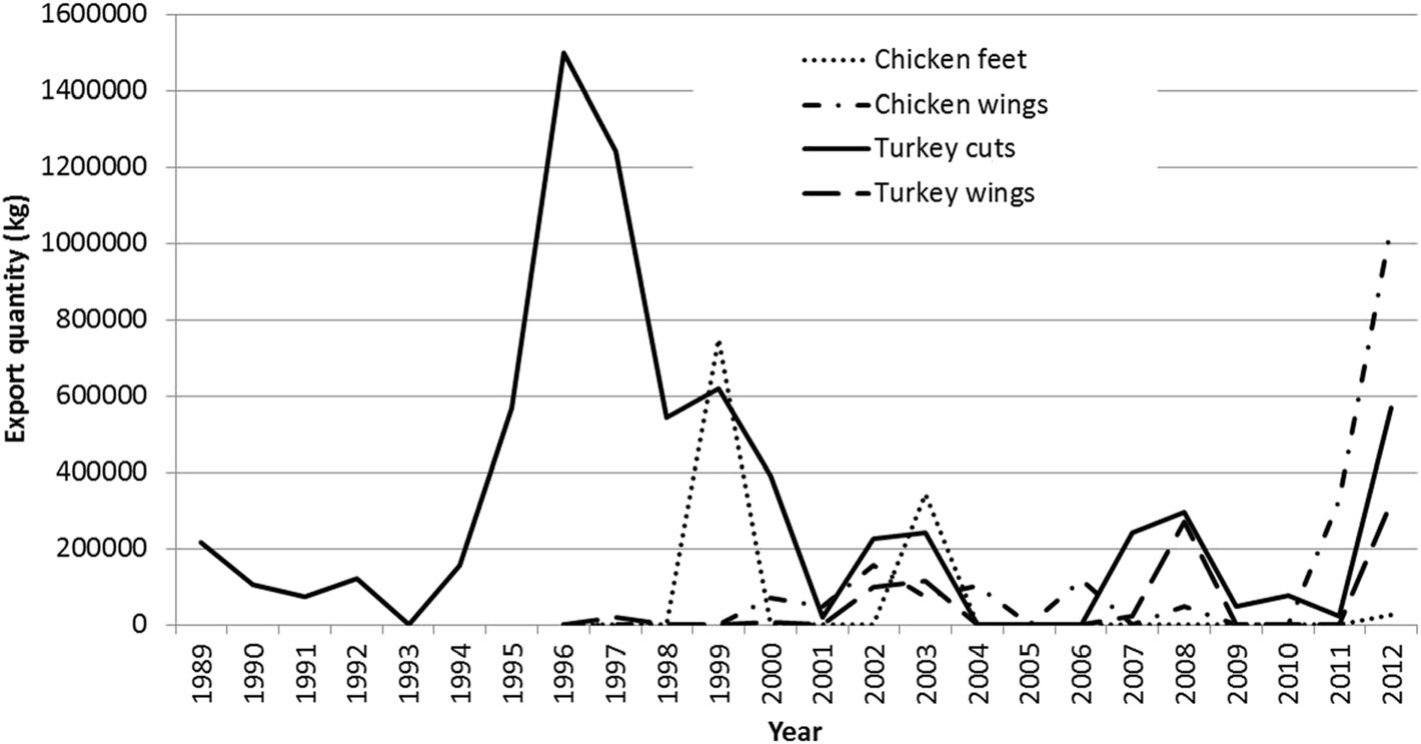
**Exports from the USA to Ghana, turkey and chicken cuts, excluding chicken leg quarters, 1989–2012.** Source: United States International Trade Commission, Interactive Tariff and Trade DataWeb (http://dataweb.usitc.gov/scripts/user_set.asp).

The overall effect of the ban has been to reduce availability of specific ‘low quality’ high-fat meats in the Ghanaian food supply, namely turkey tails and chicken feet. Detailed data for chicken imports from the USA (the main source of chicken imports until the mid-2000s) show that unspecified turkey cuts other than wings and legs (i.e. likely to be turkey tails) declined in the early 1990s with the initiation of the food standards (Figure 3). They subsequently rose in the mid-1990s, along with imports of chicken feet, and then both declined sharply after the 1999 communique issued by the Ministry of Trade, reminding the public of the standards. Imports of turkey tails rose again in 2007, and then ceased after the public concerns over implementation and subsequent highly publicised enforement measures. Imports rose again in 2011 and 2012, generating further media commentary [43].

**Figure 4:**
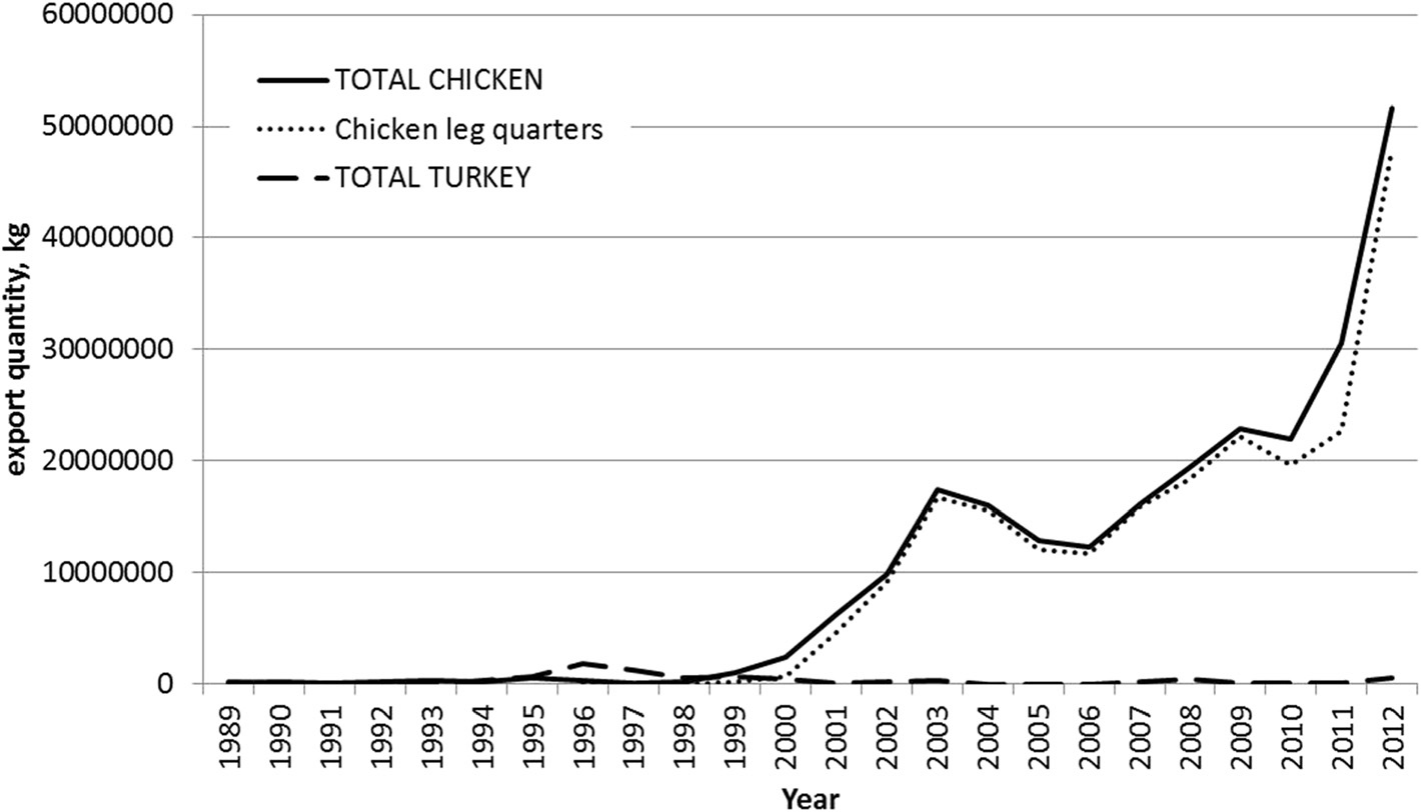
**Exports from the USA to Ghana, chicken and turkey, 1989–2012.** Source: United States International Trade Commission, Interactive Tariff and Trade DataWeb (http://dataweb.usitc.gov/scripts/user_set.asp).

Overall poultry import data from the US show that the major import is now chicken leg quarters (Figure 4). According to the United States Department of Agriculture, Grade B chicken leg quartersfrom the USA (the only information available) contain 13% fat, and would therefore comply with the standard [44]. Interviewees reported that the other main imported meat product is now offal, which is a traditionally consumed meat product and usually low in fat, and that people also eat more imported fish (depending on the season).

Interviewees from both the Department of Agriculture and from industry reported that the standards have had little effect on local agriculture. Meat production in Ghana has increased steadily [45] but supplies only around 35% of consumption due to competition from imports, which are cheaper and more convenient (e.g. poultry cuts rather than whole bird [46]). Costs of meat production in Ghana remain relatively high, largely because of the cost of feed. There is ongoing investment in agricultural extension work, but since the late 1980s the Government of Ghana has maintained minimal intervention (e.g. via subsidies or protectionism) in agriculture.

Nutritionists reported that the food environment in Ghana remains less than ideal. While there is much less turkey tail available, other sources of saturated fat include fried pork (‘domedo’) and chicken, instant noodles and sausages. Soft drinks are also heavily advertised. Diet-related NCDs continue to increase, but at the same time maternal and child undernutrition remains an issue.

## Conclusions

This study indicates that the use of standards can reduce fatty meat availability in a national food supply. In Ghana, standards have been used to substantially reduce imports of turkey tails and chicken feet; products that have also been identified as a health concern in other countries. The main strength of a standards approach to reducing fatty meat (mainly imported) in the food supply is that it is generally compliant with global trade law and much more likely than product-specific bans to be justifiable in this context. In an era of liberalized trade, global, regional and bilateral trade agreements affect all countries, and food supply-focused NCD prevention interventions must be consistent with these agreements to limit the potential for contest in these forums.

First, the standards do not discriminate between imports and domestically produced meats, and apply to the main types of meat available (both imported and local). They are thus compliant with the core trade liberalization principle of ‘non-discrimination’, which prevents countries from discriminating between goods on the basis of their country of origin, or between ‘like’ products [47]. This is a key area of difference between this approach and the use of import or sales bans to restrict imports of specific cuts of high-fat meat in Pacific Island Countries [12]. Second, the standards were developed as a response to concerns regarding human health, which is a permissible reason for implementing a measure affecting trade under WTO rules (in particular, Article XX of the General Agreement on Tariffs and Trade, and also in the Agreements on Sanitary and Phytosanitary Measures (SPS) and Technical Barriers to Trade (TBT)) [48-50].

Finally, the standards are likely to be justifiable from a technical perspective because the maximum fat percentages were based on public health concerns and applied uniformly to cuts of meat containing high levels of fats. However, a difficulty in this respect arises from the lack of an international standard regarding high-fat meats in the context of diet-related NCD prevention, as both the SPS and TBT Agreements recommend that measures be based on relevant international standards [51]. It is also not clear whether this measure falls under the TBT or SPS Agreement, and in the 2001 WTO Trade Policy Review the measure is classed as a standard (under the remit of the TBT Agreement) while in the 2008 Review it is classed as an SPS measure. This lack of clarity around high-fat meats stems from the application of the standards to an agricultural product (usually regulated by SPS), although they do not meet the formal requirements for an SPS measure, which focuses on pests and food-borne disease (for example, measures 'to protect human or animal life or health within the territory of the Member from risks arising from additives, contaminants, toxins or disease-causing organisms in foods, beverages or feedstuffs') [50]. It is thus most likely that this policy measure constitutes a TBT measure, as the Agreement on TBT defines standards and technical regulations as documents which describe 'product characteristics' [50]. The use of a specific food standard to specify the characteristics of acceptable meat with respect to fat content constitutes a key difference between this approach and product bans implemented in the Pacific Islands, which are more likely to be governed under SPS (for a detailed discussion see McGrady 2011 [52]).

This distinction has implications regarding the requirements for justifying the measure under international trade law from a technical perspective. The Agreement on TBT (Article 2) requires that measures be non-discriminatory and not more trade restrictive than necessary [50]. In relation to diet-related intervention, these requirements are less demanding than the SPS Agreement, which requires a risk assessment [52]. Given that this standard on the percentages of fat in meat is most likely to fall under the remit of the TBT Agreement, a justification for the policy could thus be based on 1) the clear relationship between the measure and the overall policy objective (to reduce consumption of high-fat meat), 2) the non-discriminatory mechanism used, and 3) the development of the policy based on a public health risk.

Our findings indicate that the main challenge in using a standards-based approach to reducing fatty meat in the food supply is effective enforcement. The maximum-fat standards in Ghana are currently not incorporated into pre-import checks, and enforcement has focussed on two specific meat cuts. This may reflect a concern that has been raised about the use of such standards in low and middle income countries: the expense of full enforcement (laboratory testing for fat content, distinguishing between individual variation in cuts etc.). However, in Ghana, the application of the standards only to cuts of meat identified as exceeding the stated percentage of fat seems to be functionally effective while maintaining low enforcement costs. This method of application still allows for adaptation to changes in food environment (e.g. using the standard to stop imports of chicken feet after they began to be imported in the mid-1990s), which makes it preferable to product-specific bans. In addition, the lack of industry complaint about the standards might be due to a possibility that such complaints could lead to broader application or more rigorous testing of imports.

It is not clear what the overall effect of the standards have been in improving diets and preventing NCDs. Ghana continues to battle a dual burden of under- and over- nutrition, and it is likely that low quality fatty meats contribute to both aspects due to their low protein content and high fat content. In a 2013 consumption survey (n = 60) conducted in Kumasi, Ghana [unpublished data from a survey conducted by the Kwame Nkrumah University of Science and Technology, used with permission], 85% participants reported avoiding fatty meat, stating that it leads to disease, and only 5% reported consuming turkey tails. Although only 15% saw the standards as reducing consumption of fatty meat, the majority (52%) had a positive outlook of the long-term effect of the restriction and supported the policy as a means to improve health and prevent disease.

Our analysis suggests two ways to strengthen the implementation and enforcement of the standards. First, Ghana could require importation report from country of origin to state fat content. Currently this is not an enforceable requirement, although in 2011 the USDA issued a new requirement for exporters to Ghana that required the labelling of poultry exports with the percentage of fat [53]. Second, it might be possible to adopt a regional approach to these standards, which would reduce cross-border trade in high-fat meat products. ECOWAS (the Economic Community Of West African States) is currently developing harmonised regional standards.

This case study offers four key opportunities for policy learning for other countries facing a rising burden of diet-related NCDs and considering the inclusion of food-supply-oriented policy approaches as part of a package of interventions (as recommended in the WHO Global Action Plan). First, the successful initiation, development and implementation of the policy required collaboration at every stage between Ministries of trade and health. Second, it is important to carefully consider and justify the policy instrument used in relation to applicable international trade law. In this case, the policy appears justifiable, but further consideration could be given to public health evidence and calculation of the requirements, keeping in mind the requirements that technical measures and standards be non-discriminatory and not more trade restrictive than necessary. Third, enforcement of the policy was enabled at a low cost by the targeting of specific commonly consumed non-compliant products. Fourth, the fluctuations in imports suggest that public awareness efforts can be an important corollary to such a policy, reflecting findings in the Pacific [12]. Regular public reminders, accompanied by periodic enforcement measures, may support the ongoing effectiveness of such food supply measures.

## Abbreviations

NCDs: Non-communicable diseases; ECOWAS: Economic Community Of West African States; EU: European Union; FAO: United Nations Food and Agriculture Organization; USA: United States of America; WTO: World Trade Organization.

## Competing interests

The authors declare that they have no competing interests.

## Authors’ contributions

AMT & RA developed the study concept and protocol, and all authors were involved in data collection and analysis. AMT drafted the paper, and all authors reviewed and approved the final version.

## Pre-publication history

The pre-publication history for this paper can be accessed here:

http://www.biomedcentral.com/1471-2458/14/249/prepub
